# Applications of graph theory in protein structure identification

**DOI:** 10.1186/1477-5956-9-S1-S17

**Published:** 2011-10-14

**Authors:** Yan Yan, Shenggui Zhang, Fang-Xiang Wu

**Affiliations:** 1 Department of Applied Mathematics, Northwestern Polytechnical University, Xi’an, Shaanxi 710072, P.R. China; 2 Division of Biomedical Engineering, University of Saskatchewan, Saskatoon, SK S7N 5A9, Canada

## Abstract

There is a growing interest in the identification of proteins on the proteome wide scale. Among different kinds of protein structure identification methods, graph-theoretic methods are very sharp ones. Due to their lower costs, higher effectiveness and many other advantages, they have drawn more and more researchers’ attention nowadays. Specifically, graph-theoretic methods have been widely used in homology identification, side-chain cluster identification, peptide sequencing and so on. This paper reviews several methods in solving protein structure identification problems using graph theory. We mainly introduce classical methods and mathematical models including homology modeling based on clique finding, identification of side-chain clusters in protein structures upon graph spectrum, and *de novo* peptide sequencing via tandem mass spectrometry using the spectrum graph model. In addition, concluding remarks and future priorities of each method are given.

## Background

Protein structure identification is a central research area in proteomics [1]. Proteins, as we know, are complex organic compounds, which consist of series of amino acids. Protein structures are usually considered as four different levels from amino acids sequences to various folding patterns. They are very important in proteomics since they usually determine the function, homology and other features of proteins. Therefore, increasing number of researchers are focusing on protein structure identification problems. Usually, biological experiments for identifying protein structures produce huge quantity of data. Facing these molecular biology data, researchers aim to find perspective relationships of proteins through effective analyzing and then, focusing on further biological relationships and functions of them [2]. In order to deal with these, biological ways have been used at first time. However, due to various limitations such as strict environment request and high experiment cost, these methods have encountered tough difficulties. Mathematical methods, by contrast, are effective in summarizing and predicting biological characteristics with lower cost, which are drawing increasing attention and being widely used in this area. Among different kinds of mathematical methods, graph theory is an essential one [3], which owns advantages in various protein structure identification problems including predicting protein structure, identification of side-chain clusters in protein structures, *de novo* sequencing, and so on [4,5].

In this paper, we summarize current applications and development of graph theory modeling in protein identification, mainly introducing three classical methods and mathematical models including homology modeling based on clique finding, identification of side-chain clusters in protein structures upon graph spectrum, and *de novo* peptide sequencing via tandem mass spectrometry using the spectrum graph model. Besides, we briefly analyze the advantages and disadvantages of these methods and give some possible directions for future research.

## Review

### Basic knowledge of graph theory

In order to understand the problem modeling, we need to know some basic concepts and background knowledge in graph theory. A *graph G* is an ordered pair (*V* (*G*), *E*(*G*)) consisting of a set *V*(*G*) of *vertices* and a set *E*(*G*), disjoint from *V*(*G*), of *edges*, together with an *incident function ψ_G_* that associates with each edge of *G* an unordered pair of vertices (not necessary distinct), if e is an edge and u and v are vertices such that ψG(e) ={u, v}, then the edge *e* is said to *join* the vertices *u* and *v*, and *u* and *v* are called the *ends* of *e*[*6*]. We denote the numbers of vertices and edges in *G* by *v*(*G*) and *e*(*G*), which are called the *order* and *size* of *G*, respectively. In this paper, we always use *G* to represent a graph we are concerning.

**Figure 1:**
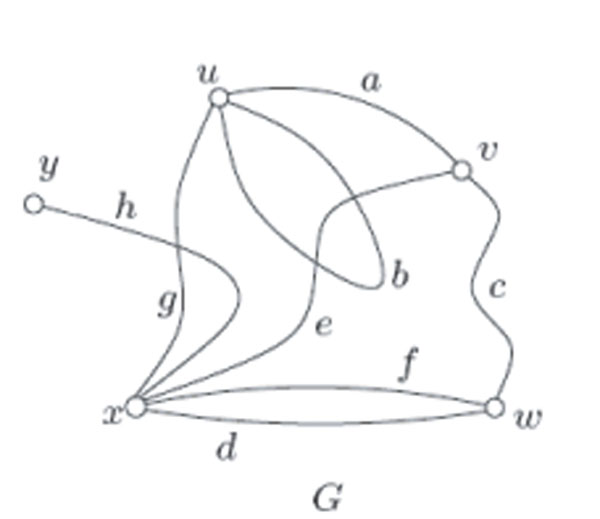
An example of graph *G*[6].

The following is an example of a graph to clarify the definition. For notational simplicity, we use *uv* for the unordered pair {*u*,*v*}. Let *G* = (*V*(*G*), *E*(*G*)), where *V*(*G*) = {*u*, *v*, *w*, *x*, *y*}, *E*(*G*) = {*a*, *b*, *c*, *d*, *e*, *f*, *g*, *h*}. The function *ψ_G_* is defined as: *ψ_G_*(*a*) = *uv*, *ψ_G_*(*b*) = *uu*, *ψ_G_*(*c*) = *vw*, *ψ_G_*(*d*) = *wx*, *ψ_G_*(*e*) = *vx*, *ψ_G_*(*f*) = *wx*, *ψ_G_*(*g*) = *ux*, *ψ_G_*(*h*) = *xy*. The graph *G* could be drawn as in Figure 1.

An edge with identical ends is called a *loop*, and an edge with distinct ends a *link* . Two or more links with the same pair of ends are said to be *parallel edges*. A graph is *simple* if it has no loops or parallel edges. In this paper, all the graphs we concern are simple graphs.

A *complete graph* is a simple graph in which any two vertices are adjacent, an *empty graph* one in which no two vertices are adjacent (that is, one whose edge set is empty). A *path* is a simple graph whose vertices can be arranged in a linear sequence in such a way that two vertices are adjacent if they are consecutive in the sequence, and are nonadjacent otherwise. The *length* of a path is the number of its edges. In a graph *G*, the *degree* of a vertex *v*, denoted by *d_G_*(*v*), is the number of edges of *G* incident with *v*, each loop counting as two edges. The set of all vertices incident with *v* is denoted by *N_G_*(*v*) [6].

In a graph, a *clique* is a set of mutually adjacent vertices, in other words, a subset of *V*(*G*) that has completely connected vertices. So in a clique, arbitrarily choosing two vertices, they are connected with each other. A clique in a graph is *maximum* if the graph contains no larger cliques. If a subgraph *S* in a graph *G* is a clique, then the clique center is a vertex *v* in *S* satisfying that, ∀*u* ∈ *V*(*S*) \ *v*, *maxd*(*u*, *v*) is minimal. The clique center is *weighted* if *G* is weighted in calculating distance.

*Adjacency matrix* of a graph *G* is the *n* × *n* matrix *A_G_* := (*a_uv_*), where *a_uv_* is the number of edges joining vertices *u* and *v*. Each loop is counted as two edges [6]. A set of points in space can be represented in the form of a graph where the points represent the vertices of the graph and the distances between the points represent edges. The constructed graph can be represented mathematically in the form of a matrix called the *Laplacian matrix*[*7*]. *Graph spectrum* is the information on analyzing the eigenvalues and eigenvectors related to Laplacian matrix in the graph spectrum research. It can gain information on cliques and clique centers in the graph.

### Construction of homology modeling upon best-weight clique finding

#### Problem description

Homology modeling is a key aspect in preteome study. When we say that sequence *A* has high homology to sequence *B*, we claim that not only sequence *A* looks much the same as sequence *B*, but also all of their ancestors look the same, going all the way back to a common ancestor [8]. Identification of homological sequences enables us to assign information from one known sequence to another unknown sequence, which enables to save lots of time and energy in research, too. However, homology modeling is facing many difficulties nowadays. One problem is that it is usually hard to find acceptable conformations of proteins because many conformations are highly dependent on experiment environment which would definitely limit the experiment design. Another problem is that there is no much effective algorithm available to cope with biological methods. Therefore, researchers are thinking of different mathematical approaches to solve these problems. Among them, the graph-theoretic method is a typical one. In this section, we will introduce a graph-theoretic method that constructs homology modeling upon best-weight clique finding. We first introduce some concepts, followed by modeling process, and then evaluate this method, giving some future research directions at last.

*Homology modeling*, also known as comparative modeling of proteins, is a technique that identifies approximate structure of a target protein from a related known homologous protein. When the target sequence is closely related to some known sequence, their overall folds are similar [9], so we can reconstruct the structure of target protein (from sequence) if we recognize its folding way by the known protein.

The steps of *homology modeling* can be arranged as follows. First, identifying an alignment between the target and related protein sequences [10]. Second, copying the main-chain coordinates from the related protein for equivalent residues and inferring some side-chain conformations. Last, building other structures left. In this procedure, current numerical methods encounter difficulties because it is hard to find suitable models [11-16]. A good model should not only satisfies the polypeptide chain property that steric exclusive effect makes energy surface discontinuous and that the conformation is context-dependent, but also has effective algorithms in implementing. Here, a graph-theoretic method can be applied to solve this problem well [17].

#### Graph-theoretic modeling

In 1998, Samudrala and Moult transferred homology modeling into a clique finding problem in graph theory and used an effective algorithm to solve it [18]. The vertices and edges of the graph are defined as follows.

*Vertex*: Each possible conformation of an amino acid residue in the sequence stands for a vertex in the graph. The *weight* of the vertex depends on interaction strength between local main-chain atoms and side-chain atoms. The main-chain atoms up to four residues on each side of the residue position, and the main-chain atoms of this residue, should be considered to calculate the weight.

*Edge:* Edges would be drawn when vertices present residue conformations within the same main-chain segment but not between clash atoms or different possible side-chain conformations of the same residue. The *weight* of an edge stands for interaction strength between two differen vertices (which represent residues).

Once the qualified graph has been drawn, all the maximal sets of cliques can be found using a clique finding algorithm [19,20]. Here, we propose an algorithm developed by Bron and Kerbosch [21].

This algorithm uses a recursive backtracking procedure and a branch-bound technique to achieve quick time clique finding [22]. There are three sets that play key roles in the algorithm: (1) *potential clique;* in this set, all the vertices are connected to each other, so this set can be extended by some new qualified vertices and has the potential to be the maximal clique. (2) *candidates;* this set consists of the vertices that can be added into the *potential clique* set. (3) *not;* this is a set of vertices that not belong to either of the former two sets, which means that the vertex has already served as an extension to the current *potential clique* set but not qualified.

At the beginning of the algorithm, *potential clique* and *not* are both empty while *candidates* consists of all the vertices of graph *G*, which represents all the possible conformations and their interactions. After that, choosing vertex *v* in *candidates* with maximal degree to the *potential clique* set. This kind of strategy makes larger cliques being found faster. Then, the vertices in *candidates* should be the vertices connected to *v*, and the vertices in *not* be the vertices disconnected to *v*. After that, choosing vertex *u* with maximal degree in the current *candidates* set, and repeating the procedure till the *candidates* set is empty. The procedure can also be written as the following steps. We use *P*, *C*, *N* to represent the sets *potential clique*, *candidates*, and *not*, respectively.

**step 1:** Set *C* = *V*(*G*), *P* = ∅, *N* = ∅;

***step 2:*** If *C* ≠ ∅, calculate , go to step 3; else go to step 4.

***step 3:****P* = *P*⋃{*v*}, *C* = *C*⋂*N_G_*{*v*}, *N* = *V*(*G*)*\*(*P*⋃*C*), go to step 2.

***step 4:*** Output *P*, stop.

Following this procedure, we can find (one of) the maximal cliques in *G*. Since each of the cliques represents a possible conformation of the sequence, the maximal one with the best weight would be considered as the most similar one to the native protein structure.

The *score* of each clique used to find maximal one with best weight is defined as(1)

where *S*(*d_ab_*) represents the score of atoms type *a* and *b* with distance *d*, *P*(*d_ab_|C*) represents the probability of observing a distance *d* between atom type *a* and *b* in a correct structure, and *P*(*d_ab_*) represents the probability of observing such a distance in all conditions without considering it is correct or not. The value of *P*(*d_ab_|C*)*/P*(*d_ab_*) is calculated by(2)

where *N*(*d_ab_*) represents the number of observations of atom types *a* and *b* in a particular distance *d*, *∑_d_N*(*d_ab_*) represents the number of *a – b* contacts observed for all distances, *∑_ab_ N*(*d_ab_*) represents the total number of contacts between all pair of atom types in a particular distance *d*, and *∑_d_∑_ab_ N*(*d_ab_*) represents the total number of contacts between all pair of atom types observed for all distances.

Given a weighted clique with *n* vertices and *m* edges representing a possible conformation, its score that represents the correctness of the probability can be calculated by(3)

where *S*(*vertex*) is the sum of the scores for distances between all atoms *p* of the side-chain and atoms *q* of the total main-chain. Therefore, we have(4)

and *S*(*edge*) is the sum of the scores for the distance between an atom *r* of one residue and an atom *s* of the other, which can be calculated by(5)

If the distance between *r* and *s* is no more than four residues, only side-chain atoms are used to calculate scores. All *S*(*vertex*) and *S*(*edge*) are calculated only once. By this means, the calculating cost can be reduced a lot.

#### Discussion and further improvement

This section gives a typical graph-theoretic method which solves homology modeling problem. It has mainly three advantages. First, it transfers a protein structure identification problem to a graph theory one, uses the algorithm of graph theory (clique finding) to solve it and makes the original problem easier to handle. Second, in this model, each score can be calculated fast, which makes the computation easy to accomplish. At last, this method excludes impossible conformation before giving weight, which eliminates the number of edges and reduces the computation scale.

However, we can also see that there are some disadvantages in this method. One is that clique finding in a given graph is an NP-hard problem that the computation time of the worst case is *O*(3*^n/^*^3^) [21], so it cannot be applied to large proteins. The other is that the function used to calculating weights of vertices and edges eliminates that the weight must be independent from other vertices and edges.

This method showed its effectiveness in the experiments done by Samudrala and Moult [18]. When the scoring function is appropriate and the CF algorithm is suitable, it can find out the native-like conformations and native structure. This method successfully calculates the fitness of a conformation, excluding a large number of unacceptable conformations, then finds the conformations represented by the cliques independently. However, if the scale of the graph is extremely large, the clique finding algorithm would be timing consuming. Further improvements of the proposed method can be focused on at least two aspects. One is improving the algorithm and the other is modifying the model. For the former one, we can try to find other advanced clique finding (CF) algorithms to reduce the computation time and broaden the range of protein size, or we may use some parallel approaches to fasten the speed. For the latter one, we can modify the original model in selection part, adding filters to exclude more unacceptable conformations to reduce the scale of the graph.

### Identification of side-chain clusters in protein structures upon graph spectrum

#### problem description

Side-chain interactions are essential to protein stability, function and folding. In protein secondary structures, the role of non-covalent side-chain interactions in stabilizing the mutual orientation has been studied well [23-25]. It is well known that clusters of hydrophobic side-chains on the surface are important for protein-protein recognition [26-30], protein oligomerization [31-33] and protein DNA interactions [34]. However, identifying side-chain interactions by experimental ways is very difficult, thus researchers prefer mathematical methods. In 1999, Kannan and Vishveswara explored a method to detect side-chain clusters in protein three-dimensional structures using a graph spectral approach [7].

#### Graph-theoretic modeling

The protein structure can be represented by a weighted graph being made up of residues. The vertices and edges are defined as follows.

*Vertex:* The *C^β^* atoms of the interacting residues are represented by vertices in a graph. Since atoms are labeled by Greek alphabetic order, *C^α^* is the carbon closest to the hydroxyl group(*–OH*), and *C^β^* is the second closest one.

*Edge:* If the distance between two *C^β^* atoms satisfies specific interaction, we draw an edge between them.

In protein structure, side-chain interactions are represented by a weighted graph and the constructed graph is represented by its Laplacian matrix. Clusters are obtained directly from the eigenvector associated with the second lowest eigenvalue of the Laplacian matrix, and the side-chains which make the largest number of interactions in a cluster (cluster centers) are obtained from the eigenvectors associated with the top eigenvalues [7]. Particularly, clustering information is sorted in the vector components of the second lowest eigenvalue, for example, all vector components in the same cluster have the same value [35], and the vector components of the top eigenvalues carry the information regarding the branching of the points forming the cluster [36] and cluster centers [37,38]. This methodology, also been used in other disciplines like electrical engineering for obtaining clusters in circuit net-lists [39], has been used here for the identification of clusters in protein structures.

An easy way to construct an adjacency matrix is to assign 1 or 0 to *a_ij_* according vertex *i* and *j* are adjacent or not in the graph. Here, we use the following weight to construct adjacency matrix.

where *d_ij_* is the distance between *C^β^* atoms of the residues *i* and *j*.

A distance of 100 is assigned to the two side-chains not satisfying the interaction criteria, hence their corresponding weight (1/100) are close to zero. The degree matrix *D* := (*d_ij_*) is constructed as:

thus, the Laplacian matrix *B* can be calculated as:(6)

Here, we also need to define a function that evaluates side-chain interactions since the definition of *A* uses it. The interaction can be calculated as(7)

where *R_i_*, *R_j_* are two different residues, *Int*(*R_i_*, *R_j_*) is the side-chain interaction of residues *R_i_* and *R_j_*, and *N*(*R_i_*, *R_j_*) is the number of all pairs of interacting side-chain atoms. Here only those atoms of residues have distance within 4.5 *Å* are calculated. *Normal*(*type*(*R_i_*)) is the normalization value of residue *R_i_* that can be calculated in advance. Here we do not concern the way of calculating this value, but only show the *Normal*(*type*(*R_i_*)) for all 20 residues (see Table 1). Detailed calculation process can be found in [7].

**Table 1:** The normal(type(*R_i_*)) for 20 residues

Residue type	Normal value
Ala	55.7551
Arg	93.7891
Asn	73.4097
Asp	75.1507
Cys	54.9528
Gln	78.1301
Glu	78.8288
Gly	47.3129
His	83.7357
Ile	67.9452
Leu	72.3517
Lys	69.6096
Met	69.2569
Phe	93.3082
Pro	51.331
Ser	61.3946
Thr	63.7075
Trp	106.703
Tyr	100.719
Val	62.3673

The following table shows the *Normal*(*type*(*R_i_*)) for 20 residues.

After that, we can define the side-chain interaction criteria in different values. Noticing that when *R_i_* and *R_j_* are fixed, *Int*(*R_i_*, *R_j_*) is fixed, too. When the side-chain interaction threshold becomes higher, fewer residues will be considered, which leads to fewer clusters being found. However, if the threshold is too low, it will result in large expanded clusters. Therefore, there is a tradeoff of setting the proper threshold in this method.

Since side-chain information can be calculated through the clique and clique center, our goal here is to find them. Specifically, Clusters are acquired from the eigenvectors associated with the second lowest eigenvalue of the Laplacian matrix, and side-chains that have the most interaction in cluster (cluster center) are acquired from the eigenvectors associated with the top eigenvalues. Therefore, the Laplacian matrix *B* contains the information of cliques and clique centers, and useful side-chains in the protein structure can be found by the above method. The detailed approach of calculating clique center upon graph spectrum and an example can be found in the Appendix of [7].

#### Discussion and further improvement

This section discusses the aspects of graph spectral approach that used for identification of side-chain clusters. Clusters are obtained directly from the eigenvectors associated with the second lowest eigenvalue of the Laplacian matrix and the side-chains which make the largest number of interactions in a cluster (cluster centers) are obtained from the eigenvectors associated with the top eigenvalues. This approach detects clusters by using different side-chain interaction criteria which can be changed by users easily. Higher side-chain interaction threshold results in less clusters while lower threshold leads to expanded clusters. Users may change the threshold to fit the specific problem they are concerning. Also, this approach can be implemented by numerical methods and the output is a simple two-dimensional cluster plot which contains the cluster and cluster center information.

However, this approach also has some disadvantages. One is that the side-chain interaction criteria is defined by researchers without any deep analysis on why this criteria is suitable, the other is that the way of constructing adjacency matrix *A* may be still simple and does not reflect interaction properly. Therefore, main issues in future can be the improvement of side-chain criteria and ways of constructing *A*.

### De novo peptide sequencing via tandem mass spectrometry

#### Tandem mass spectrometry

Nowadays, *tandem mass spectrometry* (*MS/MS*) plays an important role in protein identification problems [40,41]. It breaks a peptide into smaller fragments and measures the mass of each fragment. A typical procedure of MS/MS contains the following steps. Protein mixtures are first digested into suitable sized peptides for mass spectrometric analysis using site-specific proteases (usually trypsin). Then the peptides are ionized during a ionization process. After that, Some of the peptides are fragmented by collision-induced dissociation (CID) and their tandem mass spectra are collected then [42-45].

**Figure 2:**
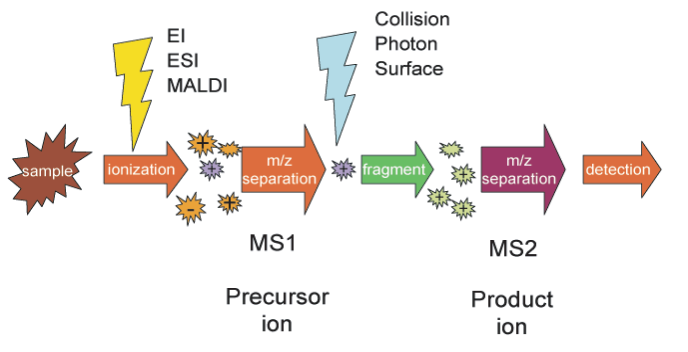
schematic of tandem mass spectrometry (from wikipedia)

A tandem mass spectrometry works like a charged sieve, we can only get a series of charged fragments from it [46,47]. Large molecules are broken into small pieces, and the problem of peptide sequencing is to find out the whole sequence of the peptide from these fragments [48]. A schematic of MS/MS is shown in Figure 2. More introduction about mass spectrometry and tandem mass spectrometry can be found in [49-54].

#### Problem of peptide sequencing

In the following subsection, we will provide the method of modeling peptide sequencing based on [5]. Let *A* be the set of amino acids, since there are 20 different amino acids in nature, *A* can be defined as:(8)

Then, the mass of each amino acid can be denoted as *m*(*a_i_*), where *i* ∈ [1, 2,…, 20].

Let *P* = *p*_1_*⋯p_n_*be a sequence of amino acids. The mass of each amino acid and the mass of parent peptide *P* are denoted as *m*( *p_i_*) and , respectively. A protein can be viewed as a chain of amino acids, which connected by a peptide bound. A peptide bound starts at a nitrogen(*N*) and ends at a carbon(*C*). We use *P_i_* to represent *N-*terminal peptide *p*_1_*⋯p_i_*, and its mass can be calculated by . Similarly, We use  to represent *C-*terminal peptide *p_i_*_+_*_1_ ⋯p_n_* with mass *m*(*P*) *– m_i_*.

When the peptide breaks down during MS/MS, it loses small pieces of molecules like water (*H*_2_*O*), *CO*–group and *NH–group*[55-57]. Assuming that there are *k* different types of ions that correspond to the removal of *k* chemical groups, the set of ions can be defined as(9)

We also use *δ_j_* to represent its mass, where *j* = 1, 2,…, *k*. A *δ – ion* of an *N*–terminal partial peptide *P_i_* is a modification of *P_i_* losing a small molecule of mass *δ*, and its mass is *m_i_* – *δ*. Similarly, we can define *δ – ion* of the *C*–terminal partial peptides [58,59].

We denote the theoretical spectrum of peptide *P* as *T*(*P*), it can be calculated by subtracting all possible ion types *δ*_1_,*δ*_2_,*…*,*δ_k_* from the masses of all partial peptide of *P*, such that every partial peptide generates *k* masses in the theoretical spectrum.

An experimental spectrum, denoted by *S*, is what we get from MS/MS, which can be defined as(10)

where *s_t_* is a fragment ion (peak) in *S*, *t* = 1, 2,…, *q*. In the following, we also use *s_t_* to represent its mass. The experimental spectrum usually includes loss of some small fragments and chemical noises. Actually, MS/MS measures *m/z* ratio, where *m* stands for mass and *z* stands for charge value (typically, it is 1, 2, or 3). Here, we assume that *z* = 1 for simplicity. The distinction of the theoretical spectrum *T*(*P*) and the experimental spectrum *S* is the mathematical results (*T*(*P*)) given the peptide sequence *P*, and the experimental spectrum (*S*) without knowing what the peptide sequence is behind this spectrum (*S*). A match of *T*(*P*) and *S* can be used to measure the relationship between the two as well as to predict peptide sequence of *S*. Therefore, the problem of peptide sequencing can be described as below.

### Problem of Peptide Sequencing

Finding a peptide whose theoretical spectrum has a maximum match to a measured experimental spectrum.

*Input:* Experimental spectrum *S*, the set of possible ion types ∆, and the parent mass *m*.

*Output:* A peptide *P* of mass *m* whose theoretical spectrum matches *S* better than any other peptide of mass *m*

#### De novo peptide sequencing method

There are mainly two ways to solve peptide sequencing problems, one is database search, and the other is *de novo* method [57,60]. The former one involves generating all 20*l* amino acid sequences of a certain length *l* and the theoretical spectrum related to each sequence, finding the maximal match among all the spectra [61-63]. Considering the number of possible sequences grows exponentially with the length of peptide sequences, the computing time would also increase exponentially. *De novo* sequencing which usually uses a spectrum graph model, on the other hand, dose not need to generate all the amino acid sequences, thus developing fast and drawing increasing attention in recent years [64-66]. Here, we introduce basic models and principles of this kind of method [5,65]. Some recent improvements and advanced approaches can be found in [67-70].

In this method, a spectrum graph representing the experimental spectrum is constructed. Assuming that experimental spectrum *S* = *s_l_*,…,*s_q_* consists of *N*–terminal ions. Here, we ignore *C*–terminal ions because we can build a similar model of *C*–terminal ions by changing *N*–terminal ions into *C*–terminal ions. Every mass of *s_t_* ∈ *S* (*t* = 1, 2,…, *q*) may have been created from a partial peptide by one of the *k* different ion types. In other words, each *s_t_* (*t* = 1, 2,…, *q*) corresponds to a spectrum of an ion, which is derived from some peptide *P_i_* (*i* = 1, 2,…, *n*) losing some small group *δ_j_* (*j* = 1, 2,…, *k*). However, we do not know what ion type of ∆ = {*δ*_1_, *δ*_2_,…, *δ_k_*} brings the mass of *s_t_*, so we need to generate *k* different *guesses* for each mass in the experimental spectrum. Every guess corresponds to a hypothesis that, let *x* be the mass of some partial peptide, then *s_t_* = *x – δ_j_*, where *t* = 1, 2,…, *q* and *j* = 1, 2,…, *k*. Therefore, there are *k* different guesses of a partial peptide with mass *x* that *s_t_* + *δ*_1_*s_t_* + *δ*_2_,…, *s_t_* + *δ_k_* corresponding to the mass *s_t_* in experimental spectrum. That is to say, a partial peptide with mass *x* has *k* different possible conformations in this model.

After that, each mass in the experimental spectrum is transferred into a set consisting of *k* vertices in spectrum graph, corresponding to each possible ion type. The problem now can be solved by using graph theory. In particular, we use a directed acyclic graph (DAG) to represent the experimental spectrum. The vertices and edges of the graph are defined as follows.

*Vertex:* Each possible conformation of a partial peptide is represented by a vertex. The vertex for *δ_j_* of the mass *s_t_* is labeled with mass *s_t_* + *δ_j_* .

*Edge:* An directed edge is drawn from vertex *u* to *v* if the mass of *v* is larger than that of *u* by the mass of a single amino acid.

Now, if we add a vertex at 0 representing the starting vertex (with mass 0) and a vertex at *m* representing the parent peptide (with mass *M*), the peptide sequencing problem can be translated into a path (from 0 to *m*) finding problem in the resulting DAG. Specifically, if there exists an edge from *u* to *v*, the chain of amino acids will be extended by adding a chemical group whose mass is the mass difference between vertex *u* and *v*. Therefore, by finding a path from 0 to *m* in the DAG, amino acid chain increases gradually and the peptide sequence can be found eventually.

In addition, vertices of the resulting spectrum graph is a set of numbers *s_t_* + *δ_j_* representing potential masses of *N*–terminal peptides adjusted by the ion type *δ_j_* . Every mass *s_t_* generates *k* different vertices, denoted by *V_t_*(*s*), then(11)

There is the possibility that *V_t_*(*s*) and *V_τ_*(*s*) may overlap when *s_t_* and *s_τ_* are close, where *s_t_*, *s_τ_* ∈ *S*. The set of vertices in a spectrum graph is therefore {*s_initial_*}⋃ *V*_1_ ⋃ ⋯ ⋃ *V_q_* ⋃ {*s_final_*}, where *s_initial_* = 0 and *s_final_* = *m*.

The spectrum graph has at most *qk* + 2 vertices. We label the edge of the spectrum graph by amino acid whose mass is equal to the mass difference between two possible conformations (vertices). If we view vertices as putative *N*–terminal peptides, the edge from *u* to *v* implies that the *N*–terminal sequence corresponding to *v* can be obtained by extending the sequence at *u* by the amino acid that labels on the edge from *u* to *v*, where *u*,*v*∈*V*(*G*).

For any *i* ∈ [1, *n*], if *S* contains at least one ion type corresponding to every *N*–terminal partial peptide *P_i_* , we say that the spectrum *S* of a peptide sequence *P* = *p_l_ … p_n_* is complete. The use of a spectrum graph is based on the fact that, for a complete spectrum, there exists a path of length *n* + 1 from *s_initial_* to *s_final_* in the spectrum graph that is labeled by *P*. This observation casts the peptide sequencing problem as one of finding the correct path in the set of all paths between two given vertices in a DAG. In addition, if the spectrum is complete, the correct path that we are finding will be the longest path in the graph usually [5].

#### Discussion and further improvement

In this section, we describe the *de novo* peptide sequencing problem and give an effective solution by a graph-theoretic method. The *de novo* method aims at inferring peptide sequences without using database, and the spectrum graph model solves this problem in a mathematical way. The solution successfully solves the problem by finding a longest path in a given spectrum graph. This kind of approach involves automatically interpreting the spectrum using the table of amino acids masses, and not relies on the completeness of database and effectiveness of searching algorithm, which the database method just relies on. Therefore, it usually costs less computation time, especially when the spectrum is with good quality.

However, this approach still has limitations. First, the success of finding the longest path in the graph relies on the completeness of mass spectrum, but in experiments, spectrum is always incomplete and combines with different kinds of noises, which makes the proposed approach hard to achieve. Second, finding the longest path in a given graph is an NP-complete problem which is difficult to find optimal solution. Third, when peptide breaks into MS/MS, it loses different kinds of small molecules, and considering all these losses needs a lot of vertices been created in the spectrum graph. When the number of vertices of the graph increases, computation time of solving this problem increases too, and even faster. At last, this kind of approach does not pay much attention to the peak intensity but using the *m/z* value only.

The performance of *de novo* peptide sequencing depends on the quality of the MS/MS spectra and the algorithms. When the spectra is complete or with high quality, *de novo* algorithm can find the correct sequences faster than the database search method, and also has the ability of finding new peptide which is not in the current database. Also, with advanced algorithm, *de novo* method could handle with spectra containing much noise, with missing peaks and so on. However, due to the limitation of tandem mass spectrometry, the database method is still the most popular and widely used one today. Some possible ways of improvements of de novo method are given below. First, when the spectrum is incomplete, we can add the missing ones by their complementary ions. Since any ion with a mass *X* in MS/MS, there should be an ion with mass *Y* such that *X* + *Y* = *M*, where *M* is the mass of the parent peptide. Thus we can add complementary ions back in an experimental spectral data set [71]. Second, we can consider effective algorithms on finding the longest path in a given graph such as dynamic programming and parallel approach. Third, this method can be partly solved by modifying the original model from finding global solution to possible local solutions. Some suboptimal algorithms can be considered, too [69]. Last but not least, a meaningful issue for the future research can be the combination of *de novo* method and other approaches, for example, database search [72].

## Conclusions

This paper reviews several methods in solving protein structure identification problems using graph theory. We first introduce the development of protein structure identification and existing problems, then giving basic knowledge of graph theory, and focusing on three typical methods using graph theory to solve protein identification problems. These methods are effective but still have problems or some inadequacy, so we also give concluding remarks of them.

In homology modeling based on clique finding, a graph that represents all the possible conformations of residues in amino acids and their interactions is drawn. We use a clique finding algorithm to find out the cliques with the best weight that are viewed as the optimal combinations of various side-chain and main-chain conformations. In identification of side-chain clusters in protein structures, graph spectral method is used. Clusters are obtained directly from the eigenvectors associated with the second lowest eigenvalue of the Laplacian matrix and the side-chains which make the largest number of interactions in a cluster (cluster centers) are obtained from the eigenvectors associated with the top eigenvalues. In *de novo* peptide sequencing via tandem mass spectrometry, the spectrum graph represents all the possible conformation of the partial peptide and the mass difference between each pair of conformations is drawn first. Then by finding the longest path in the spectrum graph, we can obtain the peptide sequence.

The above three methods all change protein identification problems into graph-theoretical ones and find effective ways of solving them. They give novel methods for handling proteomics problems and can be improved in various aspects in future. There are mainly two directions of improvements. One is the algorithm, such as improving CF algorithm and the longest path algorithm; the other is the model, for example, modifying side-chain interaction criteria. These improvements will enhance the computation ability and make the graph scale an acceptable size. We have seen that in recent literature, researchers are focusing on some of the improvements and have already done partial work successfully. However, there are still a vast amount of work for us to do to improve the current modified methods and find better ways to solve different protein identification problems in graph theoretical methods.

## Competing interests

The authors declare that they have no competing interests.

## Authors’ contributions

YY wrote the first draft of the review. SGZ intensively revised the manuscript. FXW supervised and gave suggestions of modifications of the manuscript. All authors read and approved the manuscript.
